# Prevalence of severe early childhood caries and associated socioeconomic and behavioral factors in Xinjiang, China: a cross-sectional study

**DOI:** 10.1186/s12903-017-0432-z

**Published:** 2017-12-02

**Authors:** Yan Li, Jibieke Wulaerhan, Yuan Liu, Ayinuer Abudureyimu, Jin Zhao

**Affiliations:** 1 Department of Endodontics, the First Affiliated Hospital of Xinjiang Medical University, No. 137, Li Yu Shan South Road, Urumqi, Xinjiang Province, 830054 China; 2 Stomatology Disease Institute of Xinjiang Uyghur Autonomous Region, Urumqi, Xinjiang, 830054 China

**Keywords:** Early childhood caries, Related factors, Children, Xinjiang

## Abstract

**Background:**

This study assessed the prevalence and severity of early childhood caries (ECC) and identified socioeconomic and behavioral correlates of the disease in preschool children living in Xinjiang.

**Methods:**

For this cross-sectional survey, 1727 children aged 3–5 years in Xinjiang were randomly recruited using a three-stage cluster sampling procedure. The “dmft” index according to the WHO 1997 criteria was used to assess ECC and severe ECC (S-ECC). A questionnaire was completed by caregivers. Variables included sociodemographic characteristics, dietary and oral hygiene behaviors, and access to dental services. The statistical associations of variables with ECC, S-ECC, and dmft were evaluated by univariate and multiple logistic regression analyses.

**Results:**

The prevalence of ECC was 78.2% and that of S-ECC was 41.2%; mean dmft scores were 5.61 ± 3.56 and 8.17 ± 2.94, respectively. The prevalence of ECC was significantly higher in children from Ining (OR 2.747; 95% CI 2.033–3.713), those whose caregivers had caries (OR 1.78; 95% CI 1.245–2.547), those with a dental visit in the past (OR 2.023; 95% CI 1.429–2.865), and those whose parents had received instructions on oral health care (OR 2.171; 95% CI 1.44–3.272), and increased significantly at age 4 years (OR 2.09; 95% CI 1.506–2.901) and 5 years (OR 2.666; 95% CI 1.855–3.833) and in children who starting tooth brushing at a young age (OR 1.363; 95% CI 1.171–1.587), and decreased significantly in children with a more educated mother (OR 0.817; 95% CI 0.688–1), those from high-income families (OR 0.667; 95% CI 0.582–0.765), those with low consumption of sweets (OR 0.66; 95% CI 0.57–0.763), and those who seldom ate before sleep (OR 0.557; 95% CI 0.437–0.712).

**Conclusions:**

ECC and S-ECC remain a serious problem among preschool children in Xinjiang. Caries rates were associated with sociodemographic and behavioral factors, which could be modified by public health strategies, including protection of primary dentition, extension of insurance to cover oral preventive services, improvement of the oral health care system, and public health education.

**Electronic supplementary material:**

The online version of this article (10.1186/s12903-017-0432-z) contains supplementary material, which is available to authorized users.

## Background

Early childhood caries (ECC) remains a major public health problem in preschool children, especially in developing countries, in view of its early onset and high prevalence, as well as the high likelihood of non-treatment [1–4]. ECC impacts on quality of life, increases the risk of caries in the permanent dentition, and promotes inequalities in oral health [5, 6]. Moreover, the consequences of untreated severe ECC (S-ECC) in young children are more serious than those of caries that develop in adulthood [7]. However, in most cases, ECC can be prevented, controlled, or even resolved if treated appropriately, even in the presence of S-ECC [8, 9]. Prevention of ECC is not only the responsibility of the dental profession but also that of society as a whole [5, 10]. Therefore, an effective oral health promotion strategy must be based on understanding and assessment of societal and behavioral factors potentially affecting oral health at the population level.

Societal and behavioral risk factors identified to be associated with S-ECC include low socioeconomic status, lack of fluoridation of community water, maternal factors (low education level, caries experience, less oral health knowledge), consumption of sweets, eating before sleep, inadequate dental hygiene behaviors, and lack of dental services [11–15]. These data indicate that S-ECC is concentrated among children from socially disadvantaged families and communities, those of indigenous and minority status, and those resident in less developed regions [11, 14–16].

The Xinjiang Uygur autonomous region is situated in the far northwestern part of Mainland China, and is a multi-ethnic settlement in which Uyghur and Han Chinese are the predominant ethnic groups, constituting approximately 85.4% of the total population of 2264.3 billion [17]. As a frontier province, Xinjiang is one of the less developed Chinese provinces in terms of economy and health care, where there is a total of 2573 registered dentists (including assistants) and the ratio of registered dentists to population is 1:8800. Oral health assessment and preventive programs have not been adequately implemented as yet, especially in the rural communities, which lack oral health care services [18]. There are very limited data on the prevalence of ECC and its severity among preschool children in this region. The aims of this study were to assess the prevalence and severity of ECC and to evaluate possible socioeconomic and behavioral correlates of the disease in preschool children aged 3–5 years living in Xinjiang.

## Methods

### Study population and sample size

This study was carried out in the rural and urban communities of the main region of Xinjiang. A three-stage stratified sampling method was used to recruit the study sample. In the first stage, a southern city (Kashgar) and a northern city (Ining) were chosen because they contain the largest populations in the Xinjiang region [17] (Additional file 1: Figure. S1). In the second stage, each of the two selected districts was stratified into urban and rural areas, comprising four urban districts and six rural townships in Kashgar and eight urban districts and eight rural townships in Ining. One kindergarten was randomly selected from each urban district and township. In the third stage, three kindergarten classes representing children aged 3, 4, and 5 years were randomly selected from each enrolled kindergarten. The sample size was calculated before initiation of the study assuming a prevalence of ECC of about 60% (based on an estimate of the prevalence of ECC in the Urumqi region), a margin of error of 5%, a 95% confidence level, and a 15% non-response rate. Accordingly, a sample size of 294 per age group was sought. The study was approved by the ethics committee at the First Affiliated Hospital of Xinjiang Medical University (Reference 20,130,216–103) and was carried out from March 2013 to May 2015.

### Clinical examination

To ensure reliability, four dentists were trained centrally and evaluated 20 children from a kindergarten before performing the examination until the inter-examiner and intra-examiner reliability showed a Kappa agreement of >0.85. The children’s oral health examinations were performed in the kindergarten nurses’ offices with a knee-to-knee posture between 9.30 a.m. and 11.30 a.m. under natural light using disposable dental mirrors and explorers. No radiographs were taken. The “dmft” index according to the WHO 1997 criteria was used to assess ECC and severe ECC [19]. ECC was defined as the presence of 1 or more decayed, missing, or filled tooth surfaces in any primary tooth in a child aged up to and including 71 months of age. S-ECC was defined as ≥1 decayed, missing or filled smooth surfaces in the primary maxillary anterior teeth or a dmft score ≥ 4 (age 3 years), ≥5 (age 4 years), or ≥6 years (age 5) [8]. Secondary caries was considered decayed and children suffering enamel hypoplasia were excluded. All examination records were held by the dentists.

### Questionnaire survey

The questionnaire included information on the following: sociodemographic characteristics (region, sex, age, ethnicity, residence, gestational age, family size, parental education level, average annual income, caretaker with cavities), dietary behaviors (feeding history, frequency of sweets consumption, eating before sleep), oral hygiene behaviors (start of tooth brushing, daily brushing frequency, parental supervision of tooth brushing, and using fluoride toothpaste), and use of a dental service (dental visit in the past and parents having received oral health care instruction). A questionnaire in the Uygur and Han languages was designed by the research team referencing the guidelines of the American Academy of Pediatric Dentistry and consensus in the current pediatric dental literature [8, 20], and was pre-tested for clarity. Two trained Uygur dental students instructed the caregivers on how to complete the questionnaires. Written informed consent was obtained from all caregivers who agreed to participate in the study.

### Statistical analysis

The statistical analysis was performed using SPSS version 21.0 software (IBM Corp., Armonk, NY, USA). The prevalence and mean dmft score were used to determine the extent and severity of ECC and S-ECC in the study population. Associations of variables with dmft scores were evaluated using the *t*-test and one-way analysis of variance. The χ^2^ and χ^2^ for trend were used in univariate analyses to assess the differences between the ECC and S-ECC groups. Variables that showed significant associations were included in multiple logistic regression models. A *p*-value of ≤0.05 was considered to be statistically significant.

## Results

Of 1857 children approached, 1727 were enrolled, representing a 93% response rate. The reasons for non-response were refusal to participate (33%), absence from kindergarten (50%), and failure to complete the questionnaire (17%). The study populations were evenly distributed between boys (52.1%) and girls (47.9%). The mean age of the children was 4.19 ± 0.72 years. The prevalence of ECC was 78.2% (*n* = 1351) with a mean dmft score of 5.61 ± 3.56 and that of S-ECC was 41.2% (*n* = 711) with a mean dmft score of 8.17 ± 2.94. However, 93% of the children with caries had not received treatment and 98.3% of the teeth with caries were not filled.

The prevalence and severity of ECC according to sociodemographic characteristics, dietary habits, oral hygiene habits, and use of a dental service are shown in Table 1. Results from the univariate analyses showed that 13 variables, including region (*P* < 0.001), age (*P* < 0.001), a caretaker with cavities (*P* < 0.001), mother’s education level (*P* < 0.001), father’s education level (*P* = 0.001), average annual income (*P* < 0.001), frequency of sweets consumption (*P* < 0.001), eating before sleep (*P* < 0.001), start of tooth brushing (*P* < 0.001), daily brushing frequency (*P* < 0.001), parental supervision of tooth brushing (*P* < 0.001), a dental visit in the past (*P* < 0.001), and parents having received oral health care instruction (*P* = 0.004) were associated with ECC. Ten variables, including age (*P* = 0.012), a caretaker with cavities (*P* < 0.001), mother’s education level (*P* < 0.001), father’s education level (*P* = 0.005), average annual income (*P* < 0.001), frequency of sweets consumption (*P* < 0.001), eating before sleep (*P* < 0.001), parental supervision of tooth brushing (*P* = 0.011), a dental visit in the past (*P* < 0.001), and parents having received oral health care instruction (*P* < 0.001) were associated with S-ECC. As expected, the trend of mean dmft was similar in statistical significance to that of ECC.

**Table 1:** Prevalence and univariate variable analysis of risk factors with ECC and S-ECC

Variable	N	%		ECC (%)				S-ECC (%)			dmft		
			ECC	Non-ECC	χ^2^	P-value	S-ECC	Non-S-ECC	χ^2^	P-value	Mean (SD)	t/F	*P*-value
Sociodemographic													
Region													
*Kashgar*	893	51.7	668 (74.8)	225 (25.2)	12.73	<0.001	350 (39.2)	543 (60.8)	2.981	0.084	4.25 (3.93)	−1.559	0.119
*Ining*	834	48.3	683 (81.9)	151 (18.1)			361 (43.3)	473 (56.7)			4.54 (3.88)		
Sex													
*male*	899	52.1	715 (79.5)	184 (20.5)	1.874	0.171	381 (42.4)	518 (57.6)	1.135	0.287	4.42 (3.83)	0.349	0.727
*female*	828	47.9	636 (76.8)	192 (23.2)			330 (39.9)	498 (60.1)			4.35 (3.99)		
Age (years)													
3	307	17.8	198 (64.5)	109 (35.5)	48.949^a^	0.001	104 (33.9)	203 (66.1)	8.847^a^	0.012	2.92 (3.38)	35.735^b^	<0.001
4	780	45.2	612 (78.5)	168 (21.5)			326 (41.8)	454 (58.2)			4.33 (3.83)		
5	640	37.1	541 (84.5)	99 (15.5)			281 (43.9)	359 (56.1)			5.16 (4.03)		
Ethnicity													
*Han*	718	41.6	565 (78.7)	153 (21.3)	0.154	0.694	306 (42.6)	412 (57.4)	1.065	0.302	4.56 (4.06)	1.592	0.112
*Uygur*	1009	58.4	786 (77.9)	223 (22.1)			405 (40.1)	604 (59.9)			4.26 (3.79)		
Residence													
*urban*	914	52.9	709 (77.6)	205 (22.4)	0.492	0.483	358 (39.2)	556 (60.8)	3.21	0.073	4.28 (3.93)	−1.192	0.234
*rural*	813	47.1	642 (79.0)	171 (21.0)			353 (43.4)	460 (56.6)			4.51 (3.88)		
Gestational age													
*full term*	1511	87.5	1171 (77.5)	340 (22.5)	5.351^a^	0.069	621 (41.1)	890 (58.9)	2.193^a^	0.334	4.39 (3.94)	0.502^**b**^	0.605
*premature birth*	160	9.3	130 (81.3)	30 (18.8)			62 (38.8)	98 (61.3)			4.23 (3.75)		
*post-term pregnancy*	56	3.2	50 (89.3)	6 (10.7)			28 (50.0)	28 (50.0)			4.84 (3.36)		
Family size													
*one children*	455	26.3	350 (76.9)	271 (21.3)	0.618	0.432	187 (41.1)	748 (58.8)	0.001	0.971	4.37 (3.85)	−0.308	0.758
*more than one children*	1272	73.7	1001 (78.7)	105 (23.1)			524 (41.2)	268 (58.9)			4.44 (4.06)		
Caretaker with cavities													
*no*	1355	78.5	1029 (75.9)	326 (24.1)	19.321	<0.001	511 (37.7)	844 (62.3)	31.048	<0.001	4.13 (3.85)	−5.258	<0.001
*yes*	372	21.5	322 (86.6)	50 (13.4)			200 (53.8)	172 (46.2)			5.32 (3.99)		
Mother’s education level													
*none or primary school*	247	14.3	221 (89.5)	18 (10.3)	32.636^a^	<0.001	120 (48.6)	87 (50.0)	35.465^a^	<0.001	5.01 (3.64)	9.629^b^	<0.001
*middle school*	601	34.8	485 (80.7)	126 (21.7)			261 (43.4)	336 (57.9)			4.62 (3.99)		
*high school*	520	30.1	382 (73.5)	138 (23.5)			213 (41.0)	341 (58.0)			4.45 (4.23)		
*university*	359	20.8	263 (73.3)	94 (24.4)			117 (32.6)	252 (65.5)			3.47 (3.25)		
Father’s education level													
*none or primary school*	174	10.1	156 (89.7)	26 (10.5)	15.916^a^	0.001	87 (50.0)	127 (51.4)	12.941^a^	0.005	5.13 (3.87)	7.343^b^	<0.001
*middle school*	580	33.6	454 (78.3)	116 (19.3)			244 (42.1)	340 (56.6)			4.43 (3.91)		
* high school*	588	34	450 (76.5)	138 (26.5)			247 (42.0)	307 (59.0)			4.61 (4.26)		
* university*	385	22.3	291 (75.6)	96 (26.7)			133 (34.5)	242 (67.4)			3.65 (3.20)		
Average annual income (RMB)													
*0–2999*	293	17	277 (94.5)	16 (5.5)	98.971^a^	<0.001	156 (53.2)	137 (46.8)	98.971^a^	<0.001	5.43 (3.62)	17.262^b^	<0.001
*3000–7999*	443	25.7	379 (85.6)	64 (14.4)			203 (45.8)	240 (54.2)			4.92 (3.80)		
*8000–19,999*	268	15.5	194 (72.4)	74 (27.6)			89 (33.2)	179 (66.8)			3.56 (3.59)		
*More than 20,000*	723	41.9	501 (69.3)	222 (30.7)			263 (36.4)	460 (63.6)			3.94 (4.06)		
Dietary behaviors													
*feeding history*													
*breast only*	832	48.2	647 (77.8)	185 (22.2)	0.224^a^	0.894	354 (42.5)	478 (57.5)	2.627^a^	0.269	4.41 (3.86)	0.137^b^	0.872
*breast and bottle*	775	44.9	609 (78.6)	166 (21.4)			315 (40.6)	460 (59.4)			4.39 (3.95)		
*bottle only*	120	6.9	95 (79.2)	25 (20.8)			42 (35.0)	78 (65.0)			4.21 (3.96)		
Frequency of sweets consumption													
*more than once a day*	398	23	365 (89.4)	42 (10.6)	75.719^a^	<0.001	215 (54.0)	183 (46.0)	41.272^a^	<0.001	5.38 (4.0)	21.975^b^	<0.001
*once a day*	483	28	399 (82.6)	84 (17.4)			197 (40.8)	286 (59.2)			4.61 (3.83)		
*less than once a day*	627	36.3	459 (73.2)	168 (26.8)			231 (36.8)	396 (63.2)			3.96 (3.81)		
*never*	219	12.7	137 (62.6)	82 (37.4)			68 (31.1)	151 (68.9)			2.76 (3.54)		
Eating before sleep													
*often*	139	8	133 (95.7)	6 (4.3)	50.041^a^	<0.001	83 (59.7)	56 (40.3)	32.036^a^	<0.001	5.64 (3.71)	15.995^b^	<0.001
*sometimes*	1120	64.9	895 (79.9)	225 (20.1)			472 (42.1)	648 (57.9)			4.53 (3.94)		
*no*	468	27.1	323 (69.0)	145 (31.0)			156 (33.3)	312 (66.7)			3.67 (3.76)		
Oral hygiene behaviors													
Start tooth brushing													
*3 years old or earlier*	818	47.4	594 (72.6)	224 (27.4)	49.121^a^	<0.001	322 (39.4)	496 (60.6)	5.821^a^	0.121	4.18 (4.12)	3.274^b^	0.02
*4 years old*	330	19.1	248 (75.2)	82 (24.8)			130 (39.4)	200 (60.6)			4.17 (3.77)		
*5 years old*	170	9.8	147 (86.5)	23 (13.5)			70 (41.2)	100 (58.8)			4.66 (3.57)		
*never brushed*	409	23.7	362 (88.5)	47 (11.5)			189 (46.2)	220 (53.8)			4.85 (3.67)		
Daily brushing frequency													
*at least once a day*	876	50.7	665 (75.9)	211 (24.1)			344 (39.3)	532 (60.7)			4.10 (3.85)		
*less than once a day*	588	34	453 (77.0)	135 (23.0)	19.833^a^	<0.001	243 (41.3)	345 (58.7)	5.193^a^	0.075	4.41 (3.90)	3.081^b^	0.027
*never brushed*	263	15.2	233 (88.6)	30 (11.4)			124 (47.1)	139 (52.9)			4.78 (3.77)		
Parental supervision of tooth brushing													
*no*	1140	66	921 (80.8)	219 (19.2)	12.919	<0.001	494 (43.3)	646 (56.7)	6.483	0.011	4.58 (3.86)	2.908	0.004
*yes*	587	34	430 (73.3)	157 (26.7)			217 (37.0)	370 (63.0)			4.01 (3.96)		
Using fluoride toothpaste													
*no*	1548	89.6	1214 (78.4)	334 (21.6)	0.336	0.562	635 (41.0)	913 (59.0)	0.137	0.711	4.41 (3.94)	0.793	0.428
*yes*	179	10.4	137 (76.5)	42 (23.5)			76 (42.5)	103 (57.5)			4.17 (3.56)		
Use of dental service													
Dental visit in the past													
*no*	1258	72.8	940 (74.7)	318 (25.3)	33.439	<0.001	447 (35.5)	811 (64.5)	60.775	<0.001	3.86 (3.63)	−9.364	<0.001
*yes*	469	27.2	411 (87.6)	58 (12.4)			264 (56.3)	205 (43.7)			5.79 (4.25)		
Parents received oral health care instruction													
*no*	1434	83	1103 (76.9)	331 (23.1)	8.522	0.004	557 (38.8)	877 (61.2)	18.901	<0.001	4.15 (3.78)	−5.507	<0.001
*yes*	293	17	248 (84.6)	45 (15.4)			154 (52.6)	139 (47.4)			5.52 (4.29)		

^a^χ^2^ for trend

^b^one-way analysis of variance

**Abbreviations:** *ECC* early childhood caries, *dmft* decayed-missing-filled teeth, *SD* standard deviation, *S-ECC* severe early childhood caries

Thirteen variables identified as being statistically significant in univariate analysis were entered into the logistic regression models. The results showed that the prevalence of ECC was significantly higher in children from Ining (odds ratio [OR] 2.747; 95% confidence interval [CI] 2.033–3.713), children whose caregivers had caries (OR 1.78; 95% CI 1.245–2.547), those with a dental visit in the past (OR 2.023; 95% CI 1.429–2.865), and those whose parents had received oral health care instruction (OR 2.171; 95% CI 1.44–3.272). Further, the prevalence of ECC increased significantly at the age of 4 years (OR 2.09; 95% CI 1.506–2.901) and 5 years (OR 2.666; 95% CI 1.855–3.833) and in those who started tooth brushing at a younger age (OR 1.363; 95% CI 1.171–1.587), and decreased significantly in children with a more highly educated mother (OR 0.817; 95% CI 0.688–1), those from a high-income family (OR 0.667; 95% CI 0.582–0.765), those with low-frequency sweets consumption (OR 0.66; 95% CI 0.57–0.763), and those who seldom ate before sleep (OR 0.557; 95% CI 0.437–0.712), as shown in Table 2.

**Table 2:** Risk indicators for early childhood caries in multivariate analysis

Variable	B	SE	Wald χ^2^	*P*-value	OR	95% CI	
						Lower	Upper
Constant	2.269	0.551	16.946	<0.001	9.671		
Region (reference, Kashgar)	1.011	0.154	43.230	<0.001	2.747	2.033	3.713
Age (years)			30.310	<0.001			
Age 1	0.737	0.167	19.428	<0.001	2.090	1.506	2.901
Age 2	0.981	0.185	28.045	<0.001	2.666	1.855	3.833
Caretaker with cavities (reference, no)	0.577	0.183	9.975	0.002	1.780	1.245	2.547
Mother’s education level (reference, none or primary)	−0.202	0.103	3.848	0.050	0.817	0.668	1.000
Father’s education level (reference, none or primary)	0.024	0.104	0.053	0.819	1.024	0.836	1.255
Annual family income	−0.404	0.070	33.713	<0.001	0.667	0.582	0.765
Frequency of sweets consumption (reference, more than once)	−0.416	0.074	31.236	<0.001	0.660	0.570	0.763
Eating before sleep (reference, often)	−0.584	0.125	21.960	<0.001	0.557	0.437	0.712
Start tooth brushing (reference, ≤3 years old)	0.31	0.078	15.985	<0.001	1.363	1.171	1.587
Daily brushing frequency (reference, less than once)	0.036	0.136	0.071	0.789	1.037	0.795	1.353
Parental supervision of tooth brushing (reference, no)	−0.271	0.147	3.404	0.065	0.762	0.571	1.017
Dental visit in the past (reference, no)	0.705	0.177	15.759	<0.001	2.023	1.429	2.865
Parents having received oral health instruction (reference, no)	0.775	0.209	13.717	<0.001	2.171	1.440	3.272

Age code: age of 3 (0,0); age of 4 (1,0); age of 5 (0,1)

Abbreviations: *CI* confidence interval, *OR* odds ratio, *SE* standard error

A similar result was found for S-ECC, the prevalence of which was significantly higher in children who had a caregiver with caries (OR 1.827; 95% CI 1.43–2.334), those with a dental visit in the past (OR 2.253; 95% CI 1.774–2.861), and those whose parents had received oral health care instruction (OR 1.821; 95% CI 1.358–2.441). The prevalence of S-ECC was significantly decreased in children from high-income families (OR 0.843; 95% CI 0.76–0.935), those with a low frequency of sweets consumption (OR 0.842; 95% CI 0.745–0.939), and those who seldom ate before sleep (OR 0.694; 95% CI 0.573–0.84), but no significant association was found with region, the child’s age, the mother’s education level, or when the child starting tooth brushing (Table 3).

**Table 3:** Risk indicators for severe early childhood caries in multivariate analysis

Variable	B	SE	Wald χ^2^	*P*-value	OR	95% CI	
						Lower	Upper
Constant	1.221	0.385	10.035	0.002	3.390		
Age (years)			3.611	0.164			
Age 1	0.282	0.149	3.577	0.059	1.326	0.990	1.776
Age 2	0.228	0.155	2.155	0.142	1.256	0.926	1.704
Caretaker with cavities (reference, no)	0.602	0.125	23.204	<0.001	1.827	1.43	2.334
Mother’s education level (reference, none or primary)	−0.113	0.078	2.103	0.147	0.893	0.766	1.041
Father’s education level (reference, none or primary)	−0.096	0.081	1.420	0.233	0.909	0.776	1.064
Annual family income	−0.171	0.053	10.452	0.001	0.843	0.760	0.935
Frequency of sweets consumption (reference, more than once)	−0.173	0.056	9.578	0.002	0.842	0.754	0.939
Eating before sleep (reference, often)	−0.366	0.097	14.108	<0.001	0.694	0.573	0.840
Start tooth brushing (reference, ≤3 years old)	−0.032	0.060	0.285	0.594	0.969	0.861	1.089
Daily brushing frequency (reference, less than once)	0.064	0.102	0.389	0.533	1.066	0.873	1.301
Parental supervision of tooth brushing (reference, no)	−0.210	0.120	3.051	0.081	0.811	0.641	1.026
Dental visit in the past (reference, no)	0.812	0.122	44.371	<0.001	2.253	1.774	2.861
Parents having received oral health instruction (reference, no)	0.599	0.149	16.074	<0.001	1.821	1.358	2.441

Age code: age of 3 (0,0); age of 4 (1,0); age of 5 (0,1)

Abbreviations: *CI* confidence interval, *OR* odds ratio, *SE* standard error

## Discussion

Our study found high prevalences of ECC and S-ECC (78.2% and 41.2%, respectively) among preschool children aged 3–5 years who were living in Xinjiang. The prevalence of ECC in this survey was significantly higher than that in children in mainland China overall (53.6%) in 2010–2013 [1]. The prevalence of ECC in 5-year-old children (84.5%) is not only higher than that in other surveys from adjacent provinces (71.4% in Qinghai, 73.8% in Nei Mongol, 55.8% in Gansu) [21], but also higher than that in surveys from neighboring developing countries, including Pakistan (43.7%) [22], India (58.6%) [23], and Nepal (52%) [24]. Among those studies, an S-ECC prevalence of only 13% was reported in Pakistan [23]. Our prevalence of ECC is three times higher than that in Pakistan and is similar to the high prevalence of severe caries (44.1%) reported in Northern Thailand [25]. Moreover, we found a high mean dmft of 5.61 for ECC and 8.17 for S-ECC, as well as a very low filling rate (1.7%) among local preschool children.

The current prevalence and treatment status of ECC highlight the fact that caries remains a serious and urgent problem among children in Xinjiang. In accordance with several known orcontroversial factors related to ECC and S-ECC identified in previous studies [1, 11, 12, 20], the prevalence of ECC is determined by a complex interaction between sociodemographic and behavioral factors. Lower sociodemographic status (region, less well-educated mother, low-income family, caregiver with cavities), risky dietary behavior (high-frequency sweets consumption, often eating before sleep), risky oral hygiene behavior (starting tooth brushing at an older age), and use of a dental service (dental visit in the past, parents had received oral health care instruction) were associated with ECC and S-ECC in the present study. Our study found that Ining (81.9%) and Kashgar (74.8%) children had a high prevalence of ECC and the prevalence was significantly different between the two regions. The higher caries rates of children in the two regions may reflect inequalities in socioeconomic conditions, an inadequate governmental oral health care system, limited human resources, poor behaviors, and limited awareness of oral health care measures. According to data from the Xinjiang Statistics Bureau in 2013 [17], local governmental health expenditure of 160.91 billion RMB accounted for 4.6% of the total health care budget, but the spending on oral health care was very low. Although there are no accurate statistics, it is likely that over 90% of total oral health expenditure in Xinjiang is not covered by basic medical insurance. Oral care services are paid for mainly by patients themselves. During the same year, the average annual income in Xinjiang was 13,585 RMB, and only 7296 RMB for a rural family, so most low-income families could not afford dental treatment, which may be one reason for the high caries and low filling rates in the region. People with a lower income and poor educational level usually have a relatively lower life expectancy, and children from low-income families are more likely to suffer from childhood illnesses [26]. Moreover, maternal education influences beliefs and attitudes towards the oral health care of children [15]. Mothers and caregivers play a role in cultivating children’s dental health behavior, including tooth brushing, dental care, and dietary habits, which are acknowledged to be protective factors for the primary teeth [15, 20]. Like in other studies [13, 15, 27], the present study found negative associations of a more highly educated mother and a higher average annual income with ECC, and in contrast, there was a positive correlation between caregivers with cavities and caries rates.

Furthermore, the number of registered dentists in Xinjiang was 2573 in 2013 and the dentist to population ratio was only 1:8800, which is far below the average of approximately 1:2000 in most developing countries [1]. The shortage of dentists, particularly specialists in pediatric dentistry, is another important reason for the lack of treatment available for ECC in the less developed frontier provinces of China [28]. Further, risky dietary [6, 14] and poor oral hygiene [11, 15] behaviors have been found to be strongly associated with the prevalence of ECC. As reported in previous studies, we found that a low frequency of sweets consumption, no eating before sleep, and starting tooth brushing at a younger age had a significant negative relationship with the risk for caries. Fewer public oral health education initiatives have been undertaken by government medical organizations or individuals in the two study regions. Most parents and caretakers were unaware of their children’s oral health status. Among the poorly educated Uygur parents, the language barrier added to their difficulty of accessing oral health information, which may be another reason for the severe caries status in their children. It would be helpful to provide information not only in Chinese but also in the native languages of minority groups when implementing dental health education strategies. Most parents did not focus on oral health care information until their children visited a dentist for severe toothache. Hence, we found that many children with ECC and S-ECC had made a dental visit in the past, because their parents had received oral health care instruction. Therefore, it seems that dentists do not play an effective role in caries prevention practices [11], probably because the demand for dental care is highly symptom-driven. This, together with the scarcity of dental health care services, may leave the dentist with little time and opportunity to provide preventive advice. These observations highlight the need for development of local oral health care policy to protect primary dentition, insurance reform to cover the field of oral preventive services, an improved oral health care system and health education for the public, more dentists in the rural regions, and promotion of the preventive role of dentists.

This study has some limitations, particularly its cross-sectional design, which did not allow for investigation of a cause-effect relationship. Further, only a small number of subjects were selected from more than 10,000 eligible children. We tried to minimize this potential source of selection bias by enrolling children from as many kindergartens as possible in the two study regions. Further, we cannot exclude the possibility of a degree of response bias because the data from caregivers were retrospective and caregivers may have responded with the intention of pleasing the interviewer or been guided by them during the interview. Future research on this topic should include a longitudinal study design and a larger study population.

## Conclusion

The findings of this study demonstrate that ECC and S-ECC remain a serious and urgent problem among preschool children in Xinjiang. Lower sociodemographic status (undeveloped region, a less educated mother, a low-income family, a caregiver with cavities), risky dietary behavior (high-frequency sweets consumption, often eating before sleep), risky oral hygiene behavior (starting tooth brushing at an older age), and use of a dental service (dental visit in the past, parents having received oral health care instruction) were associated with an increased risk of ECC and S-ECC. These factors could be modified by public health strategies, such as development of preventive strategies for primary dentition, reform of insurance to cover the field of oral preventive services, an improved oral health care system and health education for the public, more dentists in rural areas, and promotion of the role of dentists in prevention service.

## Additional file

Additional file 1: Figure S1. The map of Xinjiang. The red tags indicate the geographic study areas. (DOCX 64 kb) (DOCX 64 kb)

## Abbreviations

CI: Confidence interval
dmft: decayed-missing-filled teeth
ECC: Early childhood caries
OR: Odds ratio
