# Supplementary material for: Prevalence of severe early childhood caries and associated socioeconomic and behavioral factors in Xinjiang, China: a cross-sectional study
Source: BMC Oral Health. 2017 Dec 2;17:144. doi: 10.1186/s12903-017-0432-z (PMC5712104; doi:10.1186/s12903-017-0432-z)
Supplement: Additional file 1: Figure S1. — The map of Xinjiang. The red tags indicate the geographic study areas. (DOCX 64 kb) (DOCX 64 kb) [file 12903_2017_432_MOESM1_ESM.docx]

## Prevalence of severe early childhood caries and associated socioeconomic and behavioral factors in Xinjiang, China: a cross-sectional study

Yan Li^1^, Jibieke Wulaerhan^2^, Yuan Liu^1^, Ayinuer Abudureyimu^2^, Jin Zhao^1^

^1^Department of Endodontics, the First Affiliated Hospital of Xinjiang Medical University, Urumqi, Xinjiang 830054, China

^2^Stomatology Disease Institute of Xinjiang Uyghur Autonomous Region, Urumqi, Xinjiang 830054, China

**Corresponding author:** Jin Zhao

Department of Endodontics, the First Affiliated Hospital of Xinjiang Medical University, No. 137, Li Yu Shan South Road, Urumqi, Xinjiang Province 830054, China

E-mail: merryljin@sina.com

**Additional file 1**


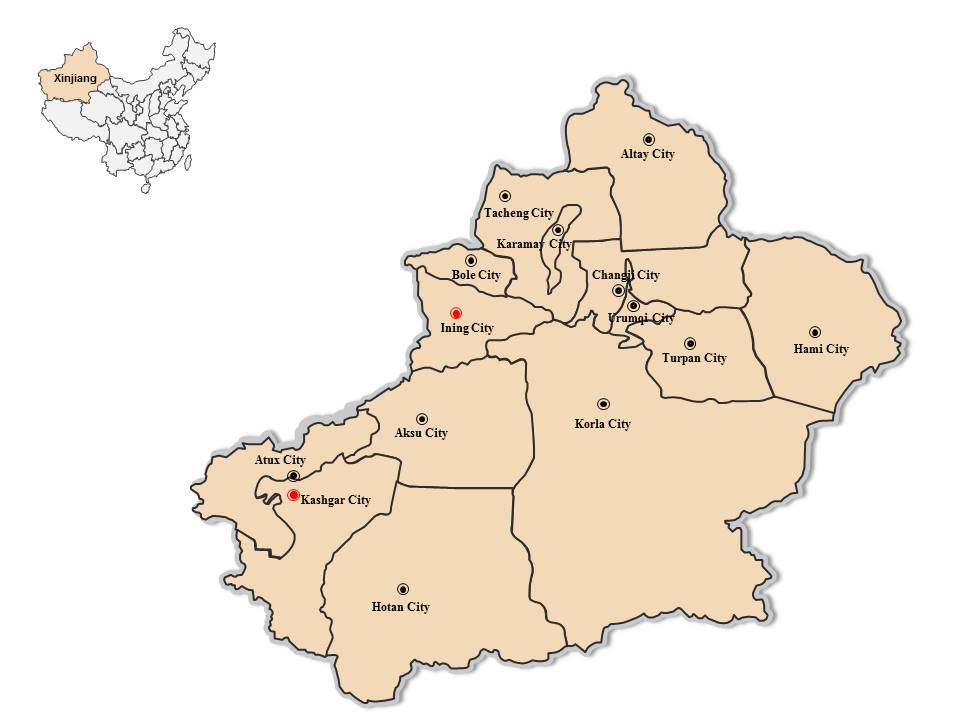


Additional file 1 **Figure S1** Map of Xinjiang. Red tags indicate the geographic study areas.
